# RNA helicase DHX15 decreases cell apoptosis by NF-κB signaling pathway in Burkitt lymphoma

**DOI:** 10.1186/s12935-021-02426-5

**Published:** 2022-02-22

**Authors:** Yuan Chen, Xianglei Chen, Lili Pan, Yuanmao Huang, Yuanhua Cai, Jinggang Li, Yang Li, Shaoyuan Wang

**Affiliations:** 1 Union Clinical Medical College, Fujian Medical University, Fuzhou, China; 2 Department of Hematology, Fujian Institute of Hematology, Fujian Medical University Union Hospital, Xinquan Road, No.29, Fuzhou, Fujian China; 3 Zhangzhou Affiliated Hospital of Fujian Medical University, Zhangzhou, P.R. China

**Keywords:** DHX15, Burkitt lymphoma, Gene knockdown, Apoptosis, NF-κB signaling pathway

## Abstract

**Background:**

DHX15 is one of the RNA helicase family members involving in several biological processes. Studies have reported that overexpression of DHX15 is related to cancer progression. However, the role of DHX15 in Burkitt lymphoma (BL) and latent Epstein-Barr virus (EBV) infection remains to be elucidated.

**Methods:**

Expression of DHX15 was measured in BL patient by immunohistochemical staining. In vitro study, a CCK-8 assay was used to analyze cell proliferation and flow cytometry was performed to assess cell cycle, apoptosis and mitochondria membrane potential. Members of NF-κB signaling pathway and apoptotic-related proteins expression were measured by western-blot. EBV latent infection products and RNA polymerase III transcripts expression were determined by quantitative real-time PCR and western-blot. In vivo study, HE, IHC, TUNEL and ISH assays were used to analyze the effect of DHX15 on subcutaneous tumor nodes formation.

**Results:**

DHX15 was overexpressed in Burkitt lymphoma patients and tends to be associated with poor progression-free survival and poor overall survival. Knockdown of DHX15 significantly inhibited BL tumor growth, reduced cell proliferation, induced cell cycle arrest and increased cell apoptosis. Further analysis showed that canonical NF-κB signaling and its downstream targets, mitochondria and Caspase were involved in the increased cell apoptosis after *DHX15* gene knockdown. Furthermore, knockdown of DHX15 reduced EBV latent infection products expression and inhibited RNA polymerase III activity.

**Conclusion:**

DHX15 may be an oncogene in the development of BL and a potential therapeutic target for the treatment of BL and latent EBV infection.

**Supplementary Information:**

The online version contains supplementary material available at 10.1186/s12935-021-02426-5.

## Background

Burkitt lymphoma (BL), first recognized as a clinical entity by Burkitt in 1958 [1], is a highly aggressive non-Hodgkin lymphoma (NHL) with extremely complex pathogenesis. All BL patients carry characteristic chromosomal translocations, resulting in constitutive expression of c-MYC protein. c-MYC is a transcription factor associated with cellular proliferation and determines cell cycle transition from G1 to S [2]. Aside from chromosome translocation, Epstein-Barr virus (EBV) also plays an important role in the development of BL. EBV was first discovered in a BL tumor from a Ugandan patient by Anthony Epstein and Yvonne Barr via electron microscopy [3]. More than 90% of BL patients are infected with EBV, most of them would enter latent infection [4, 5]. Latent EBV genomes express latent infection products, including six EBV-encoded nuclear antigens (EBNA), three latent membrane proteins (LMP), two EBV-encoded small RNA (EBER) and some microRNAs. In BL, EBV presents type I latent infection with expression of EBNA-1, EBER-1 and EBER-2, which have been found to play important roles in the development of BL [6–9].

DEAH (Asp-Glu-Ala-His) box helicase 15 (DHX15) is one of the RNA helicase family members and plays an important role in several biological aspects. First, DHX15 participates in innate immune response against viral infection by regulating several signaling pathways [10–12]. Second, DHX15 involves in modulating pre-mRNA and pre-rRNA splicing [13–17]. Third, DHX15 plays a role in further processing of RNA polymerase III primary transcripts via interaction with La (SS-B) autoantigen [18]. However, current researches of DHX15 in anti-virus mainly focused on RNA virus. It remains poorly understood whether DHX15 affects the expression of EBV latent infection products (EBNA-1, EBER-1, EBER-2) or participates in the development of BL.

Our previous study found that the *DHX15* gene was overexpressed in acute lymphoblastic leukemia (ALL) and acute myeloid leukemia (AML) patients. DHX15 was downregulated when AML patients achieved disease remission. *DHX15* gene knockdown in Jurkat and NB4 cells can induce cell apoptosis, arrest cell cycle and inhibit cell proliferation [19]. In this study, we found that DHX15 promoted cell proliferation and tumor growth, inhibited cell apoptosis, and increased the expression of type I EBV latent infection products, suggesting that DHX15 might play an important role in pathogenesis of BL and be a potential therapeutic target for treating BL.

## Material and methods

### Patient samples and follow-up

The study was approved by the Fujian Medical University Ethics Committee. Sixty-three biopsy samples preserved in Pathology Department of Union Hospital Affiliated to Fujian Medical University from January 2008 to December 2017 were obtained with written informed consent from 31 patients diagnosed with BL and 32 patients diagnosed with noncancer lymphoid reactive hyperplasia (LRH). General and clinical characteristics of BL patients were shown in Table 1. Expressions of DHX15 were detected in each specimen via immunohistochemistry (IHC). Diagnostic criteria for BL referred to World Health Organization (WHO) classification criteria for lymphohematopoietic tumors in 2008 [20].

**Table 1:** General and clinical characteristics of BL patients with high or low DHX15 expression

	NO. case	High DHX15 (%)	Low DHX15 (%)	*P*
Age				
< 14 years (%)	15	5 (33.3)	10 (66.7)	0.347
≥ 14 years (%)	16	8 (50)	8 (50)	
Gender				
Male (%)	28	11 (39.3)	17 (60.7)	0.361
Female (%)	3	2 (66.7)	1 (33.3)	
WBC count				
≥ 10 × 10^9^/L (%)	9	3 (33.3)	6 (66.7)	0.626
< 10 × 10^9^/L (%)	21	9 (42.9)	12 (57.1)	
Anemia				
Yes (%)	13	4 (30.8)	9 (69.2)	0.367
No (%)	17	8 (47.1)	9 (52.9)	
PLT count				
≥ 100 × 10^9^/L (%)	24	9 (37.5)	15 (62.5)	0.576
< 100 × 10^9^/L (%)	6	3 (50)	3 (50)	
Albumin < 35 g/L				
Yes (%)	12	3 (25)	9 (75)	0.171
No (%)	18	9 (50)	9 (50)	
LDH > 245U/L				
Yes (%)	22	9 (40.9)	13 (59.1)	0.382
No (%)	5	1 (20)	4 (80)	
UA > 420 μmol/L				
Yes (%)	15	5 (33.3)	10 (66.7)	0.597
No (%)	14	6 (42.9)	8 (57.1)	
EBER ISH ( +)				
Yes (%)	9	4 (44.4)	5 (55.6)	0.402
No (%)	8	2 (25)	6 (75)	
B symptoms				
Yes (%)	10	3 (30)	7 (70)	0.429
No (%)	20	9 (45)	11 (55)	
Stage				
I–II	5	2 (40)	3 (60)	1.0
III–IV	20	8 (40)	12 (60)	
Tumor diameter ≥ 10 cm				
Yes (%)	4	2 (50)	2 (50)	0.683
No (%)	18	7 (38.9)	11 (61.1)	

### Cell culture and lentiviruses infection

The Raji cell line and Daudi cell line were purchased from the cell library of the Chinese Academy of Medical Science and maintained at 37 °C in an atmosphere containing 5% CO_2_ in RPMI-1640 supplemented with 10% fetal bovine serum (FBS). RPMI-1640 and FBS were purchased from Hyclone company (USA) and TIAN JIN HAO YANG BIOLOGICAL MANUFACTURE CO.,LTD, respectively. DHX15-NC-Lentivirus, DHX15-shRNA-Lentivirus and polybrene were purchased from Shanghai GeneChem, China and maintained at − 80 °C. Four groups were set first (for Raji cells and Daudi cells): the blank control (CON) group, the blank control group with only polybrene at 8 μg/ml (ConP), the negative control (NC) group transfected with DHX15-NC-Lentivirus and the knockdown (KD) group transfected with DHX15-shRNA-Lentivirus. The second experiments were divided into four groups (for Raji cells solely): the CON group with only pan Caspase inhibitor Z-VAD-fmk pretreatment for 2 h at 20 μmol/L (CON + Z), the KD group transfected with DHX15-shRNA-Lentivirus with Z-VAD-fmk pretreatment for 2 h at 20 μmol/L (KD + Z), the CON and the KD group.

For cell transfection, cells were seeded in 24-well plates with 5 × 10^4^ cells per well containing 400 μl of medium for 2 h before transfection. Viral supernatants were supplemented with 8 μg/ml polybrene and incubated with target cells at a multiplicity of infection (MOI) at 80 (Raji cell line) or 120 (Daudi cell line) for 8 h. After 72 h of transfection, cells were harvested for further experiments.

### RNA extraction and quantitative real-time PCR (qRT-PCR)

Total RNA extraction was performed using TRIzol reagent (Invitrigen) according to the manufacturer’s instructions. RNA concentration was measured by ultraviolet spectrophotometer. 1000 ng of total RNA was subjected to reverse transcription to cDNA using the Verso cDNA kit (Thermo Fisher Scientific). qRT-PCR was used to quantify the expression of DHX15, EBNA-1, EBER-1, EBER-2, 5S RNA, 7SL RNA and tRNA^tyr^ in Raji cells and β-actin was used as the loading control. qRT-PCR was performed on a 7500-thermal cycle (ABI) using FastStart Universal SYBR Green Master Mix (Roche) with the following conditions: 95 °C for 2 min, 40 cycles of 95 °C for 10 s and 60 °C for 1 min. All samples were run in triplicate, and the 2^−ΔCT^ (ΔCT = CT_target gene_-CT_β-actin_) method was used to calculate the relative expression of target gene. Primer sequences were shown in Additional file 1: Table S1.

### Protein extraction and Western blot analysis

For total protein extraction, cells were washed with cold phosphate buffer solution (PBS) and subsequently lysed in cold radioimmunoprecipitation assay (RIPA) lysis buffer containing 1 mM phenylmethylsulfonyl fluoride (PMSF) and 1 mM phosphatase inhibitor on ice for 30 min. Clear protein extracts were obtained by centrifugation for 15 min at 4 °C and were quantified by ultraviolet spectrophotometer. Mitochondrial, nuclear and cytoplasmic proteins were separated from the cells according to the protocols supplied by Mitochondrial Isolation Kit for Mammalian cells and Tissues (Incent Biotechnologies, Inc), Nuclear and Cytoplasmic Protein Extraction Kit (Beyotime Biotechnology). Then, thermal denaturation of protein lysis containing 1 × SDS loading buffer was conducted at 99 °C for 10 min. 80 μg of protein mixed with SDS loading buffer was loaded per lane and separated by 12% SDS–polyacrylamide gel electrophoresis (SDS-PAGE). Proteins were transferred to nitrocellulose membrane and nonspecific binding was blocked by 5% skim milk at room temperature for 90 min. Membranes were incubated with corresponding primary antibody overnight at 4 °C. Then, membranes were washed with 1 × TBST for 10 min, three times, and then incubated with corresponding secondary antibody at room temperature for 45 min followed by washing the membrane with 1 × TBST for 10 min, three times. The immunoreactive bands were visualized using the ECL chemiluminescence detection kit for horseradish peroxidase (HRP). Images were acquired using X-ray film.

### Cell cycle assay

Cell cycle assays were performed according to the instructions of the PI/RNase Cell Cycle Detection Kit (BD) as follows: cells were washed with cold PBS twice and fixed in 500 μl 70% ethanol solution overnight at 4 °C. After that, cells were washed with cold PBS twice again and resuspended in 100 μl PI/RNase for 15 min in the dark followed by analysis of cell cycle by flow cytometry.

### Cell proliferation assay

The Cell Counting Kit-8 (CCK-8) was used for measuring cell proliferation. 7,000 viable cells per well were seeded in 96-well plates in a final volume of 100 μl. Every 24 h, a plate was subjected to measure cell proliferation by adding 10 μl of CCK-8 solution for 2.5 h (Raji cells) or 3.5 h (Daudi cells) incubation at 37 °C. The absorbance at 450/630 nm was measured by a microplate reader. The experiment was repeated three times.

### Cell apoptosis assay

Apoptosis assays were performed according to the instruction of the Annexin V-PE/7-AAD Apoptosis Detection Kit (BD) as follows: cells were washed with cold PBS twice and then resuspended in 100 μl 1 × Binding Buffer. Cells were stained with Annexin V-PE and 7-AAD for 15 min in the dark followed by measuring cell apoptosis by flow cytometry (BD).

### Mitochondrial transmembrane potential (MTP) assay

Mitochondrial transmembrane potential (MTP) assays were performed according to the manuals of the JC-1 Mitochondrial Transmembrane Potential Detection Kit (BD) as follows: cells were harvested and resuspended in 500 μl 1 × JC-1 work solution and incubated for 15 min at 37 °C. After that, cells were washed with 1 × Assay Buffer twice and resuspended in 500 μl 1 × Assay buffer followed by detecting the mitochondrial transmembrane potential by flow cytometry.

### Xenograft tumor formation

All studies on mice were conducted in accordance with the National Institutes of Health "Guide for the Care and Use of Laboratory Animals" and were approved by the Fujian Medical Experimental Animal Care Committee. Eighteen six-week-old male BALB/c nude mice were housed in a temperature-controlled, pathogen-free animal facility with a 12 h light and 12 h dark cycle in the Animal Center of Fujian Medical University. The mice were divided into three groups randomly, CON, NC and KD group, in which untransfected Raji cells, Raji cells transfected with DHX15-NC-Lentivirus or Raji cells transfected with DHX15-shRNA-Lentivirus (8 × 10^6^ cells in 200 μl/animal) were respectively subcutaneously injected into the right flank. The mice were observed twice a week and sacrificed by cervical dislocation on day 42. No anesthetic was used during the whole experiment.

### Hematoxylin–Eosin (HE) staining

Xenograft tumors were fixed in 10% neutral formalin overnight at room temperature followed by being dehydrated, transparent, embedded in paraffin and sectioned. The paraffin section of each specimen was deparaffinized, rehydrated and stained with hematoxylin and eosin according to the HE staining manufacturer’s instructions. The staining results were observed under high magnification (200 ×) using Image Pro Plus 6.0 software.

### Terminal deoxynucleotidyl transferase (TdT) mediated nick end labeling (TUNEL) immunohistochemistry analysis

TUNEL immunohistochemistry analysis was performed using the TUNEL Apoptosis Assay kit (Roche, South San Francisco, CA, US). 3 µm-thick sections were deparaffinized, rehydrated, quenched and treated with proteinase K. TUNEL immunohistochemistry analysis was performed using TdT, digoxin-labeled dUTP and a two-stage TUNEL kit according to the manufacturer’s instructions. The positive rate of each individual specimen was calculated as described above and was used to represent the apoptotic rate for an individual.

### Immunohistochemical analysis

For patient samples, paraffin-embedded specimens were collected before chemotherapy from BL and noncancer LRH patients as described above. For xenograft tumors, paraffin-embedded specimens were prepared as described above. 3 µm-thick sections were deparaffinized, rehydrated and quenched. Immunohistochemical staining was performed using primary antibodies and a two-stage immunohistochemical kit according to the manufacturer’s instructions. The number of all tumor cells and those with positive staining were calculated manually under high magnification (400 ×) using Image Pro Plus 6.0 software. Five fields were selected for each individual specimen to determine the percentage of tumor cells with positive staining among all tumor cells. The positive rate and staining intensity were used to represent the level of target protein expression. The primary antibodies against human DHX15 and Ki-67 were purchased from Abcam and EBAN-1 primary antibodies were purchased from Santa Cruz.

### *EBER *in situ* hybridization (ISH) analysis*

EBER-ISH was applied to all xenograft tumors cases using digoxin labeled oligonucleotide probes to detect the expression of EBER-1 and EBER-2. 3 µm-thick sections were deparaffinized, rehydrated, quenched, treated with pepsase and prehybridization was performed for 2 h. Sections were then incubated with EBER-1 and EBER-2 probes labeled with digoxin overnight. The next day, sections were washed with 2 × SSC, 0.5 × SSC and 0.2 × SSC successively and then incubated with monoclonal mouse anti-digoxin. An ultrasensitive ABC peroxidase mouse IgG staining kit and 3,3’-diaminobenzidine (DAB) were used for signal detection. The optical density (OD) value of each sample was calculated using Image Pro Plus 6.0 software.

### Statistical analysis

The grading data of the two groups were compared with grade two and the independent sample rank sum test (Mann–Whitney U). The data were represented as the mean ± standard deviation (X ± SD) and compared with Student’s *t* test or one-way ANOVA. The overall survival (OS) and progression-free survival (PFS) of BL patients were analyzed by the Kaplan–Meier method. All statistical analysis was performed using IBM SPSS software version 20.0 and a value of *P* < 0.05 was considered statistically significant.

## Results

### Higher expression of DHX15 in BL patients

**Fig. 1:**
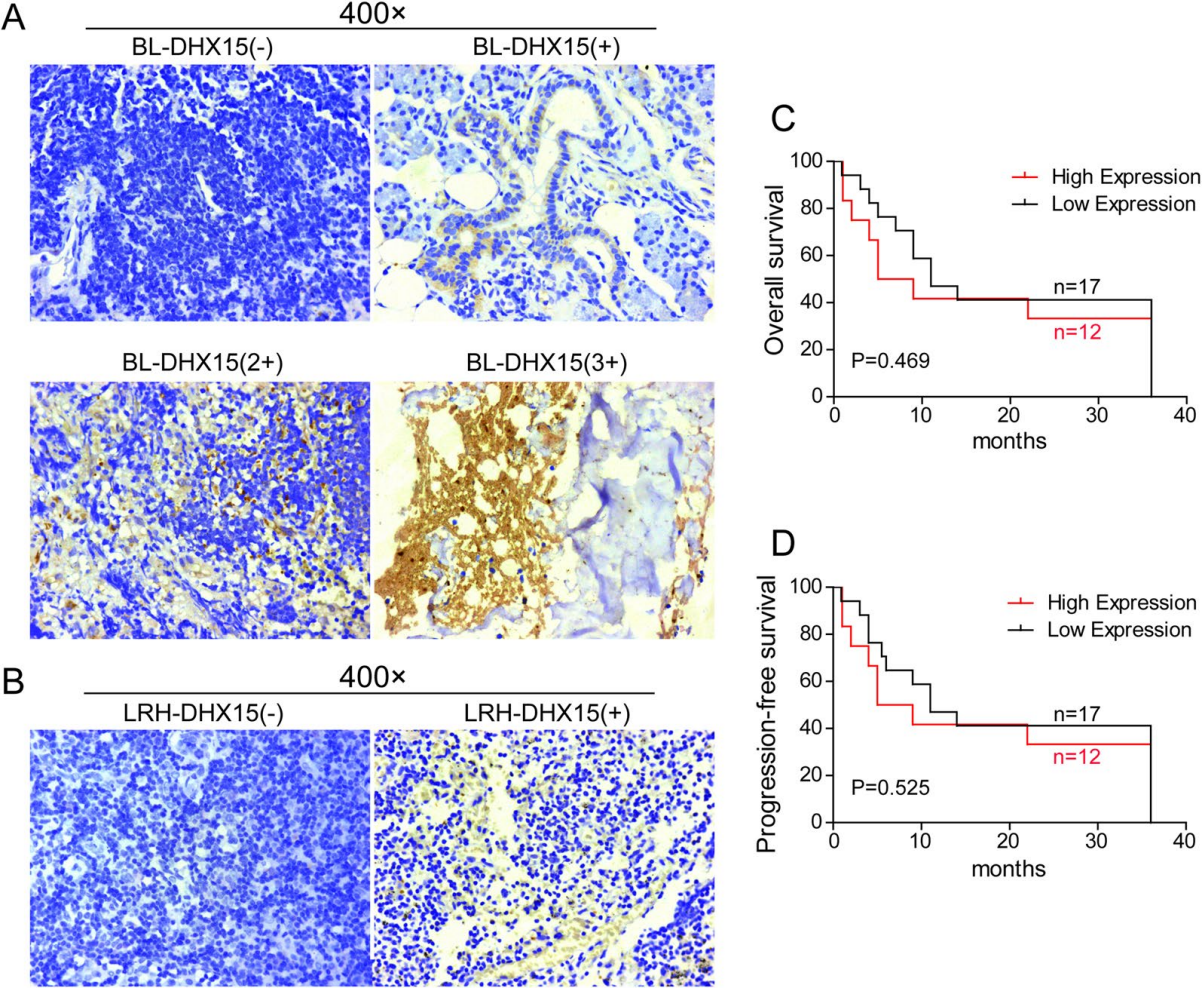
**A** DHX15 staining of tissue sections of BL patients (×400)**. B** DHX15 staining of tissue sections of noncancer LRH patients (× 400). **C** Kaplan–Meier analysis for cumulative OS curves of patients with high or low DHX15 expression. The OS time of patients with high DHX15 expression tended to shorten. **D** Kaplan–Meier analysis for cumulative PFS curves of patients with high or low DHX15 expression. The PFS time of patients with high DHX15 expression tended to shorten

To determine the expression of DHX15 in BL patients, IHC was performed and the results suggested that DHX15 expression was significantly higher in BL patients than that in the noncancer LRH patients (Figs. 1A and B, and Table 2). Then, the BL patients were divided into low (IHC positive intensity were negative or 1 +) or high (IHC positive intensity were 2 + or 3 +) DHX15 expression groups. Statistical analysis showed that there was no statistically significant difference for overall survival (OS, refers to the fact that the patient has not died from any cause) time and progression-free survival (PFS, refers to survival without progression of a particular disease) time between patients with high DHX15 expression and patients with low DHX15 expression (Fig. 1C and D).

**Table 2:** Expression of DHX15 protein in BL and noncancer LRH patients

Target protein	Type of tissue	Number of cases at all levels				Positive rate (%)	Total number	*Z*	*P*
		−	+	2 +	3 +				
DHX15	BL	10	8	6	7	67.74	31	− 4.334	< 0.001
	LRH	26	6	0	0	18.75	32		

### Silencing DHX15 downregulated the expression of EBNA-1, EBER-1, EBER-2 and RNA pol III transcripts in Raji cells

**Fig. 2:**
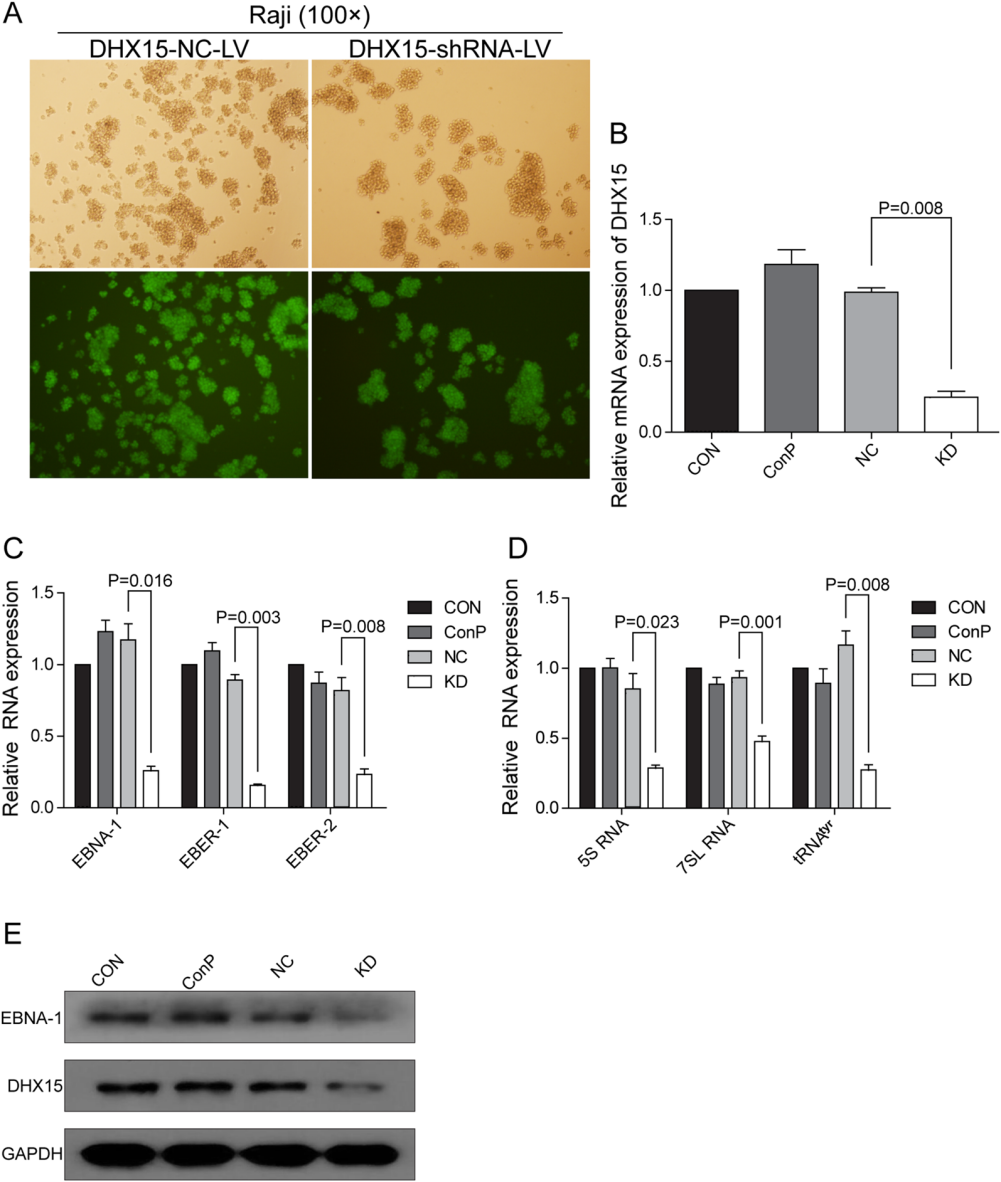
**A** Infection rate of Raji cells by NC-shRNA-LV and DHX15-shRNA-LV. The top pictures were the result of white light observation, the bottom pictures were the result of corresponding blue light excitation observation, inverted fluorescence microscope (× 100). **B** QRT-PCR verified *DHX15* gene knockdown. Compared with the NC group, *P = 0.008. **C** Effects of *DHX15* gene knockdown on the level of EBNA-1 mRNA, EBER-1 and EBER-2 in Raji cells. Compared with the NC group, *P = 0.016, **P = 0.003, ^#^*P* = 0.008. **D** Effects of *DHX15* gene knockdown on the level of 5S RNA, 7SL RNA and tRNA^tyr^ in Raji cells. Compared with the NC group, *P = 0.023, **P = 0.001, ^#^*P* = 0.008. **E** Effects of *DHX15* gene knockdown on protein level of DHX15 and EBNA-1 in Raji cells

We used lentiviral vector-mediated RNAi technique to specifically silence *DHX15* gene in Raji cells. After lentiviral transfection, most of the cells were GFP-positive in the NC and KD group, indicating a high efficiency of shRNA transfection (Fig. 2A). Lentiviral-mediated DHX15 shRNA significantly silenced *DHX15* gene expression in Raji cells compared to NC group (Fig. 2B and C). Simultaneously, the expression of EBNA-1 mRNA and protein, EBER-1, EBER-2 and RNA pol III transcripts 5S RNA, 7SL RNA and tRNA^tyr^ was decreased significantly in the KD group (Fig. 2D), indicating that the activity of RNA pol III was decreased significantly after *DHX15* gene knockdown.

### Inhibition of DHX15 induced tumor-suppressive properties in Raji cells

**Fig. 3:**
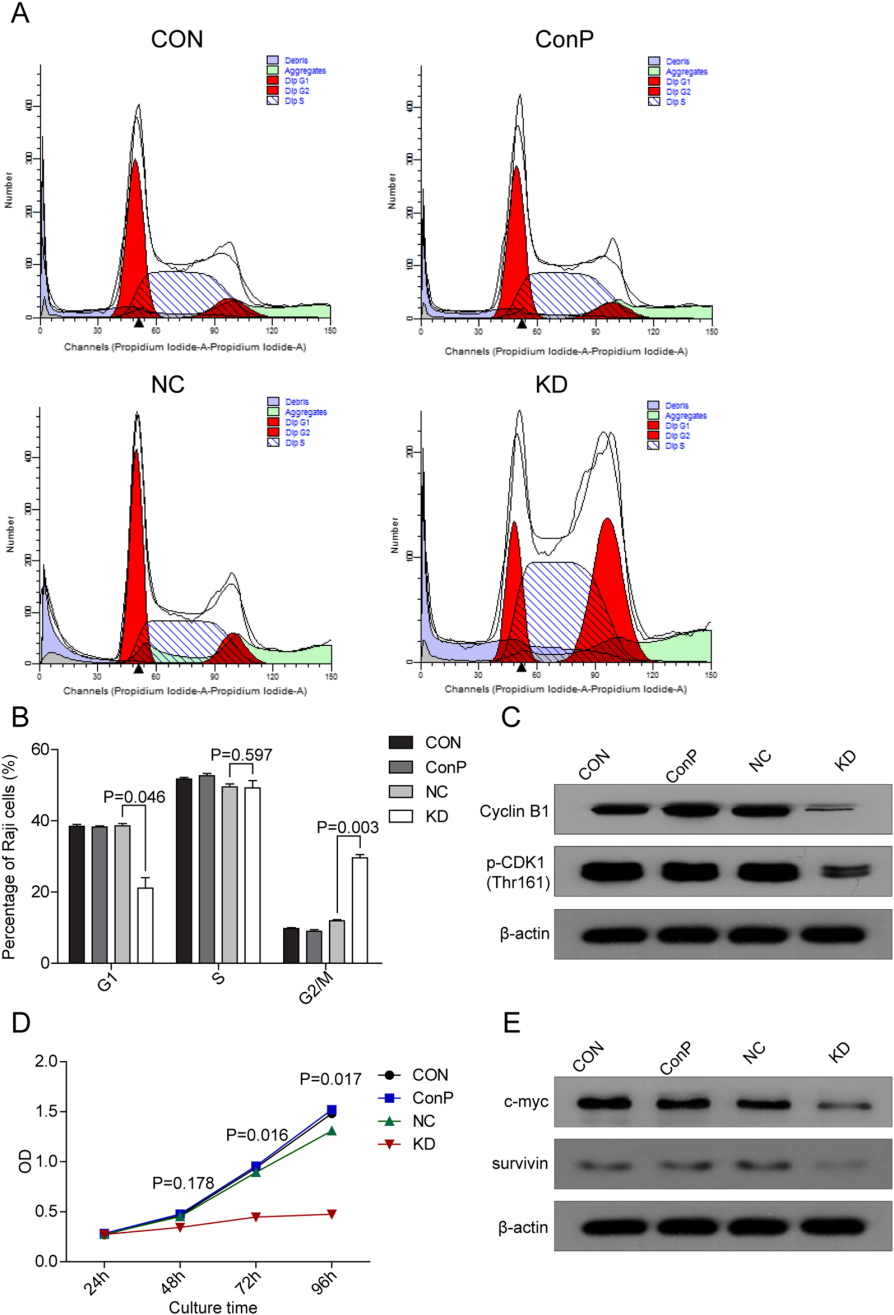
**A**, **B** Effects of *DHX15* gene knockdown on the cell cycle of Raji cells. Compared with the NC group, *P = 0.046, **P = 0.597, ^#^*P* = 0.003. **C** Effects of *DHX15* gene knockdown on the protein level of Cyclin B1 and p-CDK1 (Thr161) of Raji cells. **D** Effects of *DHX15* gene knockdown on the proliferation of Raji cells. Compared with NC group, *P = 0.178, **P = 0.016, ^#^*P* = 0.017. **E** Effects of *DHX15* gene knockdown on the protein level of c-myc and survivin in Raji cells

To study the tumor-promotive properties of DHX15 in Raji cells, cell cycle, cell proliferation and cell apoptosis were analyzed after *DHX15* gene knockdown. As shown in Fig. 3A and B, the percentage of cells at the G1 stage was significantly lower in KD group than that in NC group, and the percentage of cells at the G2 stage in KD group was significantly higher than that in NC group. These data indicated that *DHX15* gene knockdown arrested cell cycle at the G2/M phase. Further study showed that the expression of cyclin B1 and p-CDK1 (Thr161) protein, which could form maturation/mitosis-promoting factor (MPF) and promote cell cycle from G2 to M stage, was decreased significantly after *DHX15* gene knockdown (Fig. 3C).

Cell proliferation was analyzed by CCK-8 assay, results of which indicated that the OD value of KD group was significantly lower than that of NC group at 72 h and 96 h (Fig. 3D), indicating that *DHX15* gene knockdown inhibited Raji cells proliferation. Simultaneously, the expression of c-myc and survivin was decreased significantly in KD group compared to NC group (Fig. 3E).

**Fig. 4:**
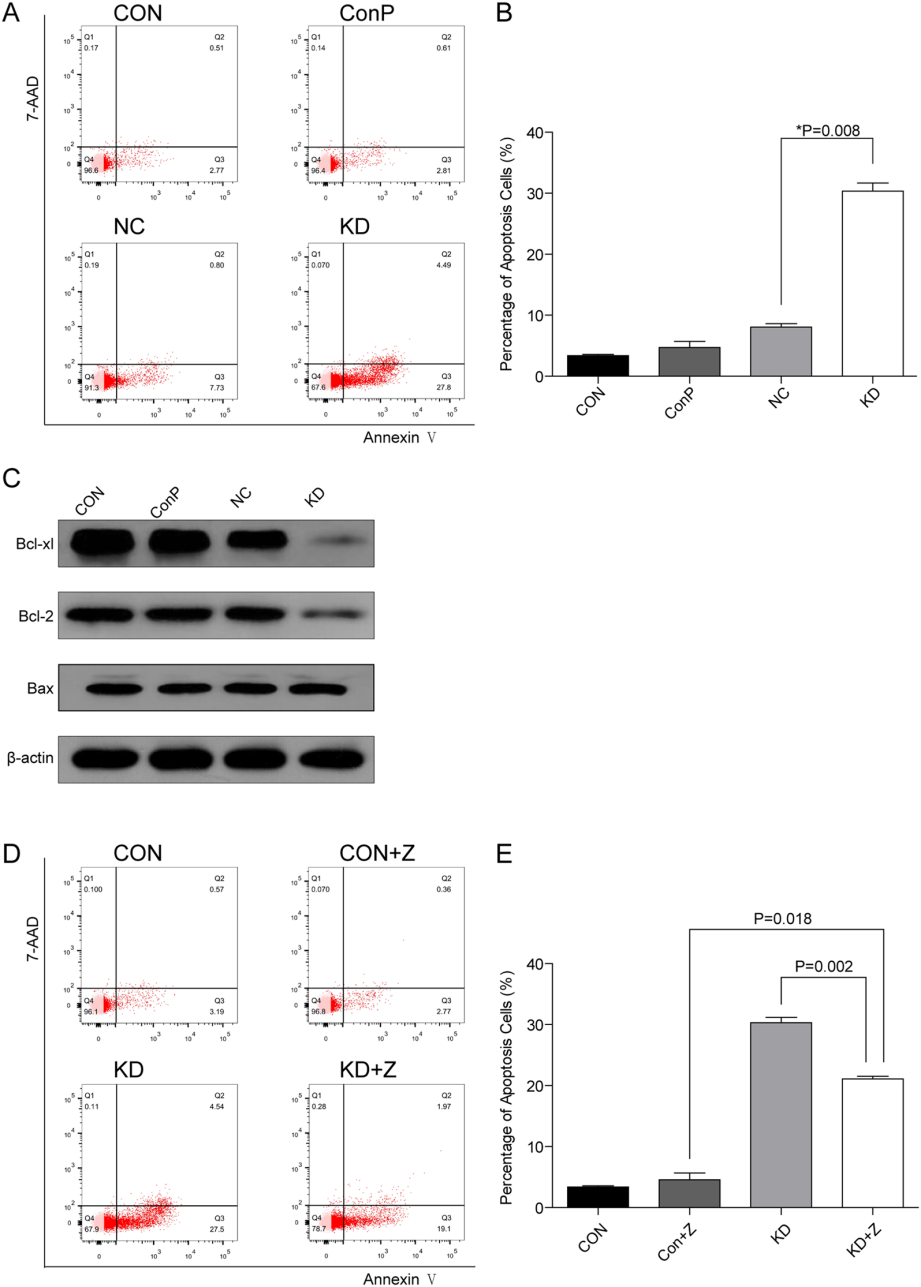
**A**, **B** Effects of *DHX15* gene knockdown on the apoptosis of Raji cells. Compared with the NC group, *P = 0.008. **C** Effects of *DHX15* gene knockdown on the level of the Bcl-2 family proteins. **D**, **E** Effects of *DHX15* gene knockdown on the apoptosis of Raji cells after Z-VAD-fmk pretreatment. Compared with the KD group, *P = 0.002, compared with CON + Z group, **P = 0.018

As shown in Fig. 4A and B, the percentage of apoptotic cells in KD group was significantly higher than that in NC group. Western Blot analysis showed that the ratio of Bcl-2/Bax, Bcl-xl/Bax were significantly decreased after *DHX15* gene knockdown (Fig. 4C). After pretreatment with Z-VAD-fmk, the percentage of apoptotic cells in KD + Z group was decreased significantly compared with KD group; however, it was still significantly higher than that in CON + Z group (Fig. 4D and E).

### Mitochondria, Caspase cascade and NF-κB signaling pathway were affected after DHX15 silencing in Raji cells

**Fig. 5:**
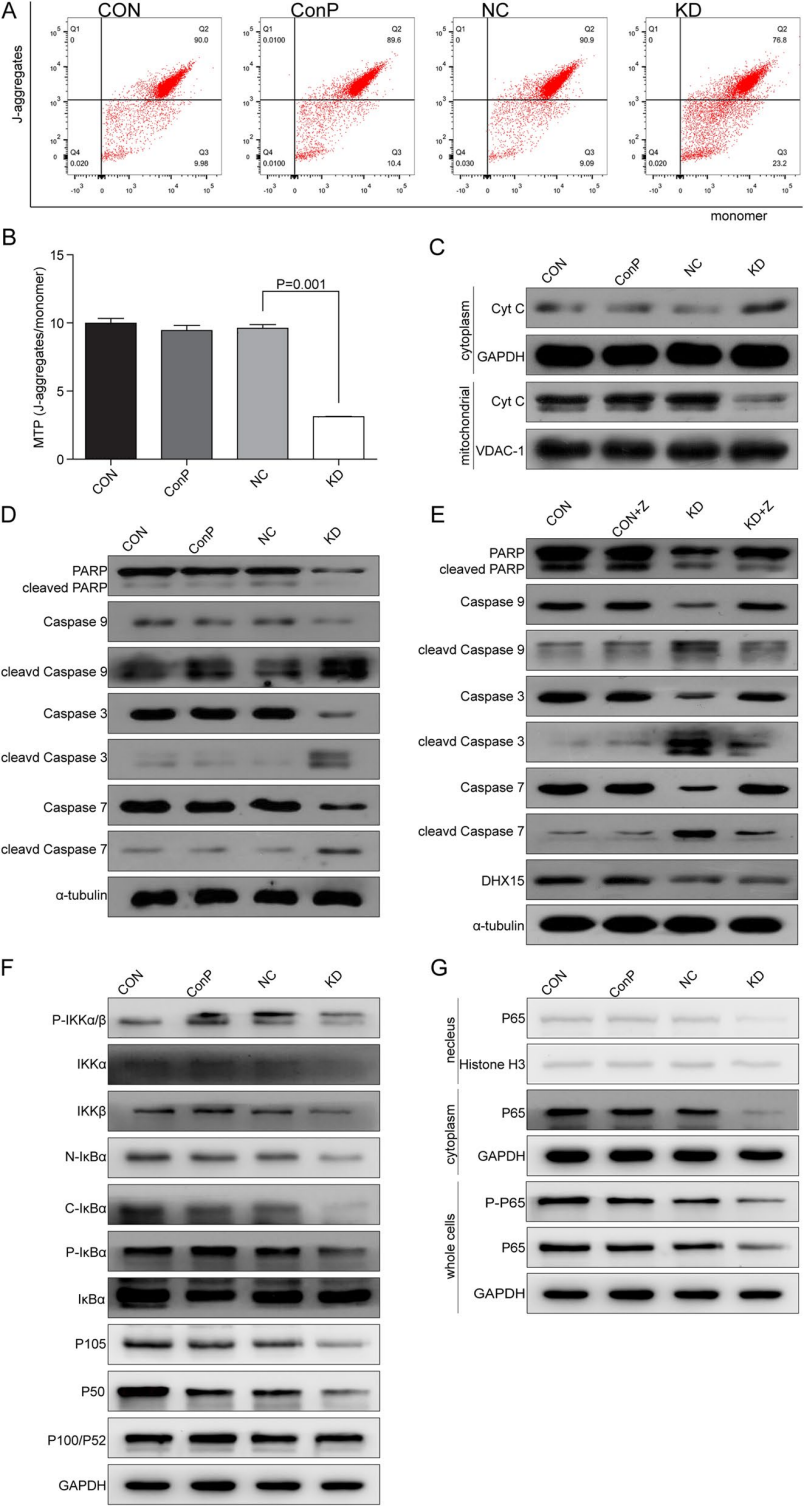
**A**, **B** Effects of *DHX15* gene knockdown on the MTP of Raji cells. Compared with the NC group, *P = 0.001. **C** Effects of *DHX15* gene knockdown on the expression of cytoplasmic cytochrome C and mitochondrial cytochrome C of Raji cells. **D** Effects of *DHX15* gene knockdown on the level of Caspase family proteins, PARP and their spliced variants in Raji cells. **E** Effects of *DHX15* gene knockdown on the level of Caspase family proteins, PARP and their spliced variants in Raji cells after Z-VAD-fmk pretreatment. **F** Effects of *DHX15* gene knockdown on the level of P-IKKα/β, IKKα, IKKβ, N-IκBα, C-IκBα, P-IκBα, IκBα, P105, P50, P100/P52 and P-P65. **G** Effects of *DHX15* gene knockdown on the level of nuclear P65, cytoplasmic P65, and overall P65

To determine the role of mitochondria, Caspase cascade and NF-κB signaling pathway in the apoptosis induced by DHX15 silencing, MTP, mitochondrial apoptotic pathway and NF-κB signaling pathway were analyzed. As shown in Fig. 5A and B, the percentage of cells with higher MTP in KD group was significantly lower than NC group and the percentage of cells of lower MTP in KD group was significantly higher than NC group, indicating that *DHX15* gene knockdown induced the decrease of MTP. Further study showed that the expression of mitochondrial cytochrome C was also decreased significantly and the expression of cytoplasmic cytochrome C was increased significantly after *DHX15* gene knockdown, indicating that cytochrome C was released from mitochondria to cytoplasm (Fig. 5C).

As shown in Fig. 5D, the expression of Caspase 9, Caspase 3, Caspase 7 and PARP was decreased significantly and their corresponding cleaved variants were increased except cleaved PARP after *DHX15* gene knockdown. After pretreatment with Z-VAD-fmk, the expression of Caspase 9, Caspase 3, Caspase 7 and PARP in the KD + Z group was increased significantly and their corresponding cleaved variants was decreased except cleaved PARP without differences compared to KD group (Fig. 5E), indicating that the Caspase cascade participates in the apoptosis after *DHX15* gene knockdown.

As shown in Fig. 5F and G, the expression of overall P65, phosphorylated P65, nuclear P65 and cytoplasmic P65 was significantly decreased after knockdown of *DHX15* gene, indicating that the P65 protein synthesis, activation and translocation into nucleus were inhibited. In addition, a significant decreased in the expression of P-IKKα/β, IKKα, IKKβ, N-IκBα, C-IκBα, P-IκBα occurred as *DHX15* gene was downregulated, indicating that the activity of IKK, being responsible for catalyzing IκBα phosphorylation, was inhibited, which finally led to reduced IκBα degradation. Moreover, the expression of P105 and P50 was decreased significantly with no change of P100/P52 expression after knockdown of *DHX15* gene, suggesting that the synthesis and activation of P105 protein was reduced.

### Silencing DHX15 downregulated the expression of EBNA-1, EBER-1, EBER-2 in Daudi cells

**Fig. 6:**
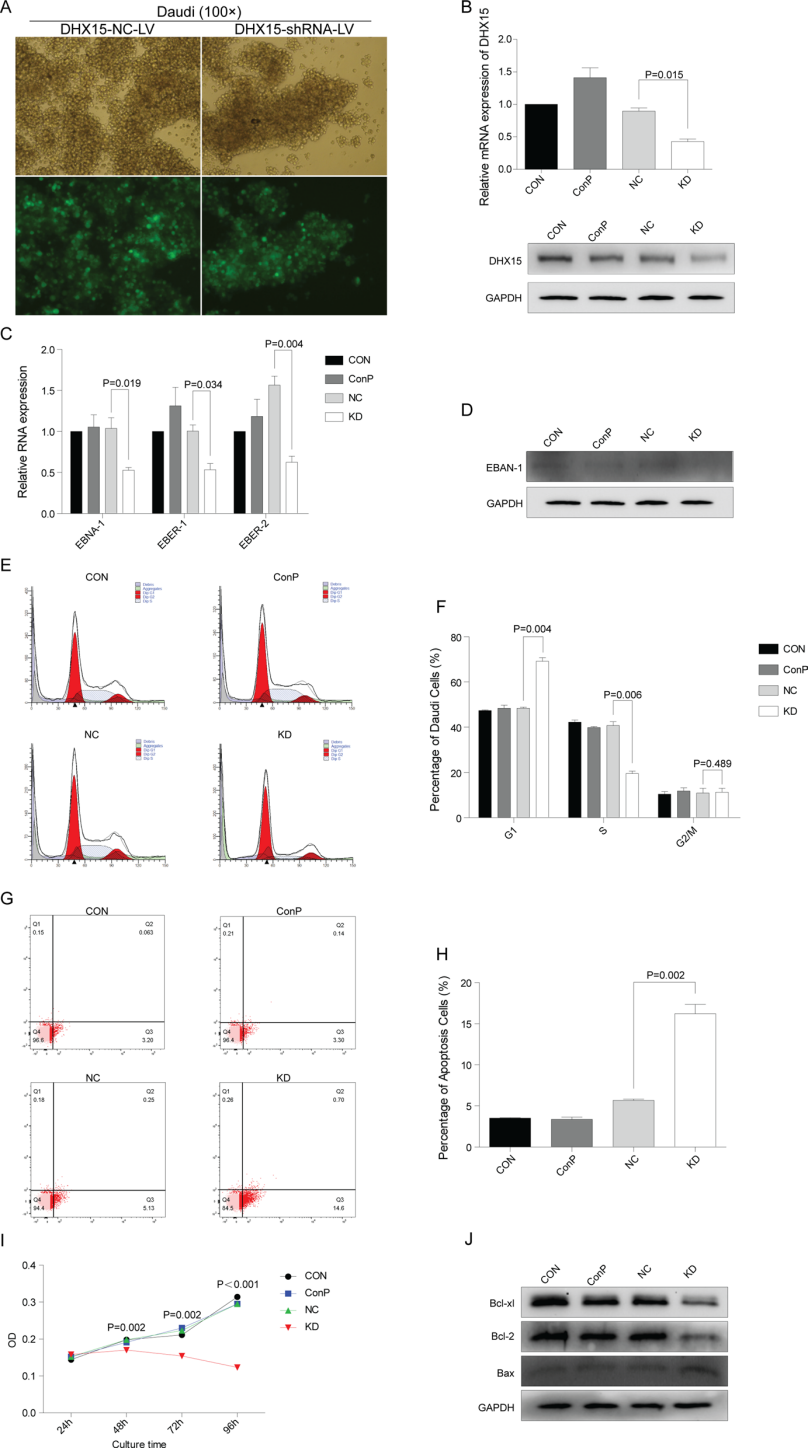
**A** Infection rate of Daudi cells by NC-shRNA-LV and DHX15-shRNA-LV. The top pictures were the result of white light observation, the bottom pictures were the result of corresponding blue light excitation observation, inverted fluorescence microscope (× 100). **B** QRT-PCR and Western-blot verified *DHX15* gene knockdown. Compared with the NC group, *P = 0.015. **C** Effects of *DHX15* gene knockdown on the level of EBNA-1 mRNA, EBER-1 and EBER-2 in Daudi cells. Compared with the NC group, *P = 0.019, **P = 0.034, ^#^*P* = 0.004. **D** Effects of *DHX15* gene knockdown on the level of EBNA-1 protein. **E**, **F** Effects of *DHX15* gene knockdown on the cell cycle of Daudi cells. Compared with the NC group, *P = 0.004, **P = 0.006, ^#^*P* = 0.489. **G**, **H** Effects of *DHX15* gene knockdown on the apoptosis of Daudi cells. Compared with the NC group, *P = 0.002. **I** Effects of *DHX15* gene knockdown on the proliferation of Daudi cells. Compared with NC group, *P = 0.002, **P = 0.002, ^#^*P* < 0.001. **J** Effects of *DHX15* gene knockdown on the level of the Bcl-2 family proteins in Daudi cells

We used lentiviral vector-mediated RNAi technique to specifically silence *DHX15* gene in Daudi cells. After lentiviral transfection, most of the cells were GFP-positive in the NC and KD group, indicating a high efficiency of shRNA transfection (Fig. 6A). Lentiviral-mediated DHX15 shRNA significantly silenced *DHX15* gene expression in Daudi cells compared to NC group (Fig. 6B). Simultaneously, the expression of EBNA-1 mRNA and protein, EBER-1, EBER-2 was decreased significantly in the KD group (Fig. 6C and D).

### Inhibition of DHX15 induced tumor-suppressive properties in Daudi cells

To study the tumor-promotive properties of DHX15 in Daudi cells, cell cycle, cell proliferation and cell apoptosis were analyzed after *DHX15* gene knockdown. As shown in Fig. 6E and F, the percentage of cells at the G1 stage was significantly higher in KD group than that in NC group, and the percentage of cells at the S stage in KD group was significantly lower than that in NC group. These data indicated that *DHX15* gene knockdown arrested cell cycle at the G1 phase.

As shown in Fig. 6G and H, the percentage of apoptotic cells in KD group was significantly higher than that in NC group. Western Blot analysis showed that the ratio of Bcl-2/Bax, Bcl-xl/Bax were significantly decreased after *DHX15* gene knockdown (Fig. 6J).

Cell proliferation was analyzed by CCK-8 assay, results of which indicated that the OD value of KD group was significantly lower than that of NC group at 48 h, 72 h and 96 h (Fig. 6I), indicating that *DHX15* gene knockdown inhibited Daudi cell proliferation.

### DHX15 silencing inhibited in vivo BL xenograft tumor formation

**Fig. 7:**
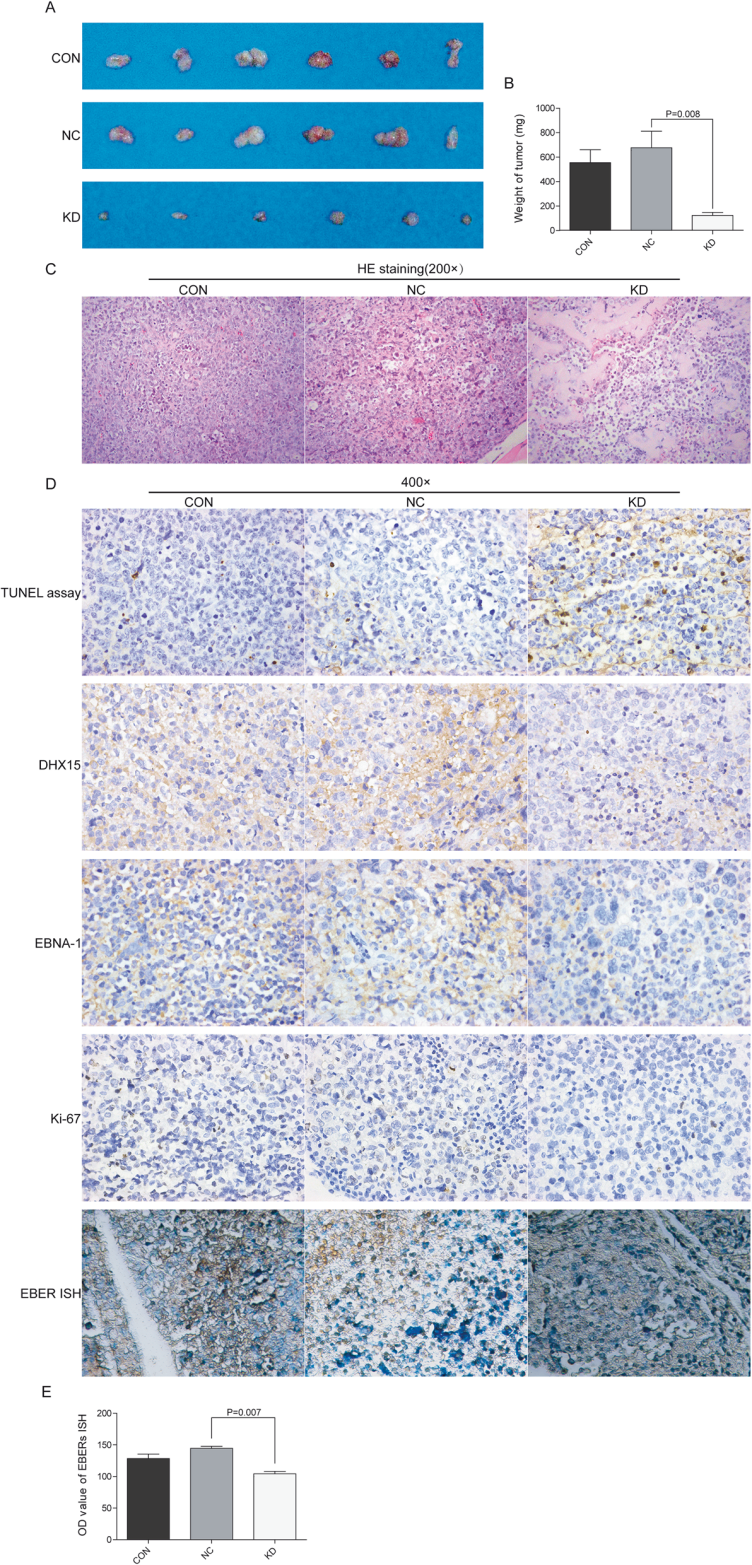
**A** General view of transplanted tumor in each group. **B** Comparison of tumor quality in each group. Compared with the NC group, *P = 0.008. **C** Routine HE staining of tumor tissues in each group (×200). **D** TUNEL assay was used to detect apoptosis in tumor tissues (400 ×). IHC detected DHX15, EBNA-1 and Ki-67 protein in tumor tissues of each group. The expression of DHX15 and EBNA-1 protein can be detected in both the cytoplasm and nucleus (×400). The Ki-67 protein was located in the nucleus (×400). Results of EBER in situ hybridization in tumor tissues of each group (×400). **E** Comparison of OD values of EBER in situ hybridization in tumor tissues of each group. Compared with the NC group, *P = 0.007

To further study the effects of *DHX15* gene knockdown on the tumorigenic phenotype of BL and its contribution to tumor growth in vivo, we successfully established a xenograft model of human BL. All nude mice could be detected with subcutaneous transplanted tumor growth. The photographic image of xenograft tumors dissected from the nude mice was shown in Fig. 7A. The xenograft tumors in the KD group were significantly smaller and lighter than that in the CON and NC group (Fig. 7B, Additional file 1: Table S2). These data indicated that *DHX15* gene knockdown inhibited xenograft tumors growth in vivo.

### HE staining

Routine HE staining was conducted after tissue section. As shown in Fig. 7C, the tumor cells in the CON group were closely aligned, with larger cell size, larger nuclei and deeper staining, and fewer cytoplasm. The tumor cells in the NC group were slightly looser than the CON group. The cell volume was larger, the nucleus was larger and deeper, the cytoplasm and blood vessels were red, and a small amount of necrotic tissue was found in the NC group. In the KD group, the tumor cells in the transplanted tumor tissue were arranged sparsely and the nuclei were narrowed. The chromatin was assembled, condensed, thickened and dyeing deepened. The apoptotic bodies appeared and many apoptotic cells and necrotic foci were found. These data indicated *DHX15* gene knockdown inhibited xenograft tumors growth and promoted apoptosis in microanatomy.

### Suppression of DHX15 induced apoptosis in vivo

TUNEL IHC analysis was performed to determine the apoptosis of xenograft tumors in each group. As shown in Fig. 7D and Table 3, the proportion of cells with nucleus staining yellowish-brown in the KD group was significantly higher than the CON and NC group, indicating that *DHX15* gene knockdown promoted apoptosis of Raji cells in xenograft tumors.

**Table 3:** Comparison of TUNEL results in tumor tissues of each group

Group	n	Positive Rate (%)	*F*	*P*	Score	*F*	*P*
CON	3	5.87 ± 2.06			1.00 ± 0		
NC	3	5.00 ± 2.85			1.00 ± 0		
KD	3	14.53 ± 4.38	7.926	0.021	1.00 ± 0	–	–

### *Suppression of DHX15 downregulated EBNA-1, EBER-1, EBER-2 and Ki-67 *in vivo*.*

IHC was performed to determine protein levels of DHX15, EBNA-1 and Ki-67 of xenograft tumors in each group. As shown in Fig. 7D and Table 4, the positive rate of DHX15 and EBNA-1 and their corresponding IHC integral in the KD group were significantly lower than the CON and NC group. The positive rate of Ki-67, which reflected the cell proliferation activity, in the KD group was also significantly lower than the CON and NC group, but there was no significant difference of IHC integral between the three groups (Fig. 7D, Table 4).

**Table 4:** Comparison of DHX15, EBNA-1, Ki-67 protein positive rate and IHC score in tumor tissues of each group

Target protein	Group	n	Positive rate (%)	*F*	*P*	IHC score	*F*	*P*
DHX15	CON	3	23.37 ± 6.39			1.33 ± 0.58		
	NC	3	30.73 ± 5.20			2.67 ± 1.15		
	KD	3	2.23 ± 3.87	26.350	0.001	0.33 ± 0.58	6.167	0.035
EBNA-1	CON	3	50.70 ± 22.11			4.67 ± 1.15		
	NC	3	48.57 ± 12.19			3.33 ± 2.31		
	KD	3	11.37 ± 10.64	5.864	0.039	0.67 ± 0.58	5.333	0.047
Ki-67	CON	3	35.80 ± 4.37			2.67 ± 1.15		
	NC	3	34.50 ± 5.90			2.00 ± 0.00		
	KD	3	18.20 ± 8.10	7.239	0.025	2.00 ± 1.73	0.308	0.746

EBER-ISH analysis was performed to determine the expression of EBER-1 and EBER-2 of xenograft tumors in each group. As shown in Fig. 7D and E, the OD value, which was proportional to the EBER level in tumor tissue, in KD group was significantly lower than that in CON and NC group. These data indicated that *DHX15* gene knockdown inhibited the expression of type I EBV latent infection products in vivo.

## Discussion

In this study, we firstly detected overexpression of DHX15, a member of the DEAH-box RNA helicase family in BL patients. Then we explored the effect of *DHX1*5 gene knockdown on BL both in vivo and in vitro. In the meantime, it is the first time to study the relationship between DHX15 and EBV. In accordance with our previous study, *DHX15* gene knockdown significantly induced cell apoptosis and cell cycle arrest, inhibited cell proliferation and growth of subcutaneous transplanted tumors in BL cells.

The transcription factor NF-κB is a key player in the inflammation, cancer development and progression [21, 22]. Aberrant NF-κB activation is a characteristic of various human malignances [21, 23]. Activated NF-κB can stimulate cell proliferation, prevent apoptosis, and promote tumor angiogenesis, epithelial-to-mesenchymal transition (EMT), invasiveness, as well as metastasis [24, 25]. Previous studies have found that constitutive NF-κB activation was involved in the pathogenesis of BL and NF-κB seemed to be required for the constitutive activation of c-myc and the upregulation of c-myc [26–28]. In our experiments, we found that *DHX15* gene knockdown inhibited the canonical NF-κB signaling pathway possibly via the following aspects: (1) inhibiting the synthesis and phosphorylation of p65/RelA protein, (2) inhibiting IκB kinase (IKK) to reduce the phosphorylation and proteasome-mediated degradation of IκBα, (3) inhibiting the activation of p105/NF-κB1 protein. Finally, *DHX15* gene knockdown inhibited the homodimer or heterodimer formation of p65 with p50, leading to reduced translocation into the nucleus and subsequent inhibition of the transcription of target genes. We also found that there was no significant change in p100/NF-κB2 protein level, a member of the non-canonical NF-κB signaling pathway. However, whether *DHX15* gene affects the non-canonical NF-κB signaling pathway is unclear and requires further study.

Several studies reported that DHX15 activates p38 MAPK and NF-κB signaling pathway during anti-virus infection [11, 12]. In our study, we found that the activity of NF-κB signaling pathway and its downstream targets, including Bcl-2, Bcl-xl, survivin, were downregulated after *DHX15* gene knockdown, indicating that *DHX15* gene knockdown may affect the function of mitochondria via Bcl-2 family members. Subsequent studies confirmed the hypothesis that MTP was decreased and cytochrome C was released from mitochondria to cytoplasm, which activated the mitochondrial apoptotic pathway leading to Raji cells apoptosis. The above results suggested that mitochondria and Caspase cascade are involved in apoptosis after *DHX15* gene knockdown in Raji cells. What’s more, we also found that the apoptosis rate of the Z-VAD-fmk pretreatment group was significantly higher than that of control group. The reasons we speculate are as follows: First, there may be other pathways that participate in cell apoptosis besides Caspase cascade, such as apoptosis inducing factor (AIF) signaling pathway [29, 30], Bcl-2 inhibitor of transcription 1 (Bit1) signaling pathway [31]. Second, the combination of the inhibitor and its substrate has a saturation effect, and Z-VAD-fmk cannot inhibit Caspase activity completely, which is also the cause of the higher cell apoptosis rate in the Z-VAD-fmk pretreatment group than that in control group. In addition, whether exogenous apoptotic pathways Caspase 8 or Caspase 10 participates in apoptosis needs to be further studied.

EBV, belonging to a family of human herpesviruses, contributes to life-long latent infection in B lymphocytes after primary infection [32]. The virus is associated with various human malignancies, such as BL, nasopharyngeal carcinoma and Hodgkin lymphoma, which could be detected in almost all samples of endemic BL patients [33]. In most BL patients, EBV shows type I latent infection with expression of EBNA-1, EBER-1, EBER-2 and BART microRNAs [34]. Previous studies had confirmed that EBV latent infection products EBNA-1, EBER-1 and EBER-2 were closely related to the occurrence and development of BL, and they could promote BL cell proliferation and inhibit BL cell apoptosis [6–9]. In our study, we found that the expression of EBNA-1, EBER-1, EBER-2 and RNA polymerase III transcripts 5S RNA, 7SL RNA and tRNA^tyr^ are downregulated after *DHX15* gene knockdown, which indicated that DHX15 may participate in the regulation of the expression of EBER-1 and EBER-2 via RNA polymerase III. However, there are no direct approaches to detect the activity of RNA polymerase III. In this experiment, we indirectly estimated the activity of RNA polymerase III by the level of specific transcripts of RNA polymerase III. Therefore, the methodology of direct detection of RNA polymerase III activity needs to be further evaluated. In a word, DHX15 may participate in the occurrence and development of BL via regulation of the expression of the above EBV latent infection products.

Moreover, we found that *DHX15* gene knockdown inhibited tumor growth and downregulated EBNA-1, EBER-1, EBER-2 in vivo. The tumor volume and weight of KD group were significantly smaller and lighter than those of the CON and NC group. Our results demonstrated that DHX15 could promoted tumor growth and upregulated EBV latent infection products.

In this study, we revealed that, compared with patients with low DHX15 expression, the overall survival time and progression-free survival time of patients with high DHX15 expression tended to shorten, but there was no significant difference. The reasons we speculate are as follows: First, the number of patients was relatively small because of low incidence rate. Second, the observation time was insufficient. Third, the patients in the group had a long-time span with different treatment options and compliance, for example, in the early years, patients with poor efficacy mostly used the CHOP chemotherapy regimen.

In summary, silencing *DHX15* gene could promote BL cells apoptosis, inhibit cell proliferation in vitro and BL tumor growth in vivo, indicating that DHX15 might be a novel therapeutic target of the treatment for BL. However, there are still some limitations in our study. For example, we did not determine whether DHX15 could also promote the expression of EBV latent infection products in other EBV-associated tumors or whether DHX15 can be used as a target for treatment of latent EBV infection. Further studies are required to explore the underlying mechanisms.

## Conclusion

*DHX15* was overexpressed in patients with Burkitt lymphoma and downregulation of *DHX15* gene promoted BL cells apoptosis, inhibited cell proliferation and BL tumor growth in vivo, suggesting that *DHX15* might be a novel therapeutic target for the treatment of Burkitt lymphoma.

## Clinical perspectives

Burkitt lymphoma (BL) is a highly aggressive non-Hodgkin lymphoma and of which Epstein-Barr virus (EBV) and constitutive expression of c-MYC protein play an important role in the development.

Knockdown of DHX15 could inhibit BL tumor growth, cell proliferation as well as the expression of latent EBV infection products and c-MYC protein.

DHX15 might be a novel therapeutic target for the treatment of BL and latent EBV infection.

## Supplementary Information

**Additional file 1: Table S1.** Oligonucleotide primers utilized in RT-PCR. **Table S2.** Tumor weight and tumor weight inhibition rate of mice in each group.

## Data Availability

Y. Chen and S.Y. Wang had full access to all the data in the study (available upon data specific request). Although all our data is de-identified, we opt not to share the data and materials in public due to further study on this subject. However, we will share the data in request by other researchers if necessary. All of the methods including the software programs or reagents used in this study are on the market, which are accessible by other researchers.

## Abbreviations

BL: Burkitt lymphoma
PFS: Progression free survival
OS: Overall survival
EBV: Epstein-Barr virus
NHL: Non-Hodgkin lymphoma
EBNA: EBV-encoded nuclear antigen
EBER: EBV-encoded small RNA
LMP: Latent membrane protein
ALL: Acute lymphoblastic leukemia
AML: Acute myeloid leukemia
LRH: Lymphoid reactive hyperplasia
IHC: Immunohistochemistry
MOI: Multiplicy of infection
PBS: Phosphate buffer solution
qRT-PCR: Quantitative real-time PCR
CCK-8: Cell counting kit-8
OD: Optical density
MTP: Mitochondrial transmembrane potential
NF-κB: Nuclear factor κB
IκB: Inhibitor of κB
N-IκBα: Amino-terminal IκBα
C-IκBα: Carboxy-terminal IκBα
IKK: IκB kinase
HE: Hematoxylin–Eosin
TdT: Terminal deoxynucleotidyl transferase
TUNEL: TdT mediated nick end labeling
ISH: In suit hybridization
