# Supplementary material for: RNA helicase DHX15 decreases cell apoptosis by NF-κB signaling pathway in Burkitt lymphoma
Source: Cancer Cell Int. 2022 Feb 22;22:92. doi: 10.1186/s12935-021-02426-5 (PMC8862312; doi:10.1186/s12935-021-02426-5)
Supplement: Supplementary file 1 — Additional file 1: Table S1. Oligonucleotide primers utilized in RT-PCR. Table S2. Tumor weight and tumor weight inhibition rate of mice in each group. [file 12935_2021_2426_MOESM1_ESM.docx]

**Additional Material**

**Additional file 1: Table S1.** Oligonucleotide primers utilized in RT-PCR

| **Gene** | **RT-PCR primer oligonucleotides (5’-3’)** |
| --- | --- |
| DHX15 | Forward: CGCATCATTGCCAAACTTC |
|  | Reverse: AAGGCCATTATCGAACCAAC |
| β-actin | Forward: AGTGTGACGTGGACATCCGCAAAG |
|  | Reverse: ATCCACATCTGCTGGAAGGTGGAC |
| EBNA-1 | Forward: CGGGATCCGGAAGGAACCATGGCCA |
|  | Reverse: GAATTCTCCTGTCATTTCATAGATCCA |
| EBER-1 | Forward: AGGACCTACGCTGCCCTAGA |
|  | Reverse: AAAACATGCGGACCACCAGC |
| EBER-2 | Forward: AGGACAGCCGTTGCCCTAGTGGTTTCG |
|  | Reverse: AAAAACAGCGGACAAGCCGAATACC |
| 5S RNA | Forward: TTTACGGCCACACCACCCTG |
|  | Reverse: AAAGCCTTCAGCACCCTGTA |
| 7SL RNA | Forward: GTGTCCGCACTAAGTTCGGCATCAATATGG |
|  | Reverse: TATTCACAGGCGCGATCCCACTACTGATC |
| tRNA^tyr^ | Forward: CCTTCGATAGCTCAGCTGGTAGAGCGGAGG |
|  | Reverse: CGGAATTGAACCAGCGACCTAAGGATGTCC |

**Additional file 1: Table S2.** Tumor weight and tumor weight inhibition rate of mice in each group

| Group | n | Tumor weight (mg) | Tumor weight inhibition rate (%) |
| --- | --- | --- | --- |
| CON | 6 | 554.7±261.7 |  |
| NC | 6 | 677.0±330.0 |  |
| KD | 6 | 121.1±62.50 | 82.11 |
